# Reprogramming MHC specificity by CRISPR-Cas9-assisted cassette exchange

**DOI:** 10.1038/srep45775

**Published:** 2017-04-04

**Authors:** William Kelton, Ann Cathrin Waindok, Theresa Pesch, Mark Pogson, Kyle Ford, Cristina Parola, Sai T. Reddy

**Affiliations:** 1 Department of Biosystems Science and Engineering, ETH Zürich, Basel, Switzerland

## Abstract

The development of programmable nucleases has enabled the application of new genome engineering strategies for cellular immunotherapy. While targeted nucleases have mostly been used to knock-out or knock-in genes in immune cells, the scarless exchange of entire immunogenomic alleles would be of great interest. In particular, reprogramming the polymorphic MHC locus could enable the creation of matched donors for allogeneic cellular transplantation. Here we show a proof-of-concept for reprogramming MHC-specificity by performing CRISPR-Cas9-assisted cassette exchange. Using murine antigen presenting cell lines (RAW264.7 macrophages), we demonstrate that the generation of Cas9-induced double-stranded breaks flanking the native MHC-I H2-Kd locus led to exchange of an orthogonal H2-Kb allele. MHC surface expression allowed for easy selection of reprogrammed cells by flow cytometry, thus obviating the need for additional selection markers. MHC-reprogrammed cells were fully functional as they could present H2-Kd-restricted peptide and activate cognate T cells. Finally, we investigated the role of various donor template formats on exchange efficiency, discovering that templates that underwent *in situ* linearization resulted in the highest MHC-reprogramming efficiency. These findings highlight a potential new approach for the correcting of MHC mismatches in cellular transplantation.

Genome engineering approaches offer tremendous potential for developing advanced cellular immunotherapies for cancer, viral infection, and hereditary disease^1^,^2^. Much of this promise is attributed to the rapid development of site-specific programmable nuclease systems, such as zinc-finger nucleases (ZFNs), transcription activator-like effector nucleases (TALENs), and clustered regular interspaced palindromic repeat Cas9 (CRISPR-Cas9)^3^,^4^. These programmable nucleases enable the targeted generation of DNA double-stranded breaks (DSB), which promote the upregulation of cellular repair mechanisms, resulting in either the error-prone process of non-homologous end joining (NHEJ) or homology-directed repair (HDR), the latter of which can be used to integrate exogenous donor DNA templates. In the context of immunotherapy, ZFNs have been used for NHEJ-induced knockout of the HIV entry co-receptor CCR5 in CD4+ T cells, a strategy that has shown promise in clinical trials^5^. CRISPR-Cas9 has also shown potential in the removal of latent HIV infection in T cells by targeting viral LTR regions, which leads to disruption or deletion of viral genes^6^,^7^,^8^. Although typically much less efficient than knock-out approaches, the use of HDR for DNA integration allows for yet more sophisticated immunotherapy applications. For example, the delivery of Cas9 protein along with oligonucleotide donor templates led to the efficient generation of point mutations in the immunomodulatory PD-1 gene in primary human T cells^9^. Additionally, a hybrid TALEN-meganuclease system was recently used to promote high-efficiency HDR integration of HIV-resistant CCR5 alleles at the native locus in human T cells^10^.

While it is evident that targeted nucleases can be used to induce NHEJ or HDR integration in immune cells, their use to mediate exchange of entire genes, particularly in polymorphic immune loci is more recent^11^. In past, the exchange of entire genes (or cassettes) relied on the use of site-specific recombinase systems such as Cre/loxP or Flp/FRT^12^. Despite being immensely valuable for the engineering of cellular and transgenic model systems, recombinase-mediated cassette exchange (RMCE) cannot be used in therapeutic settings since it requires the targeted genomic region to have pre-existing recombinase-specific sites^13^. An alternative strategy for cassette exchange, which may be amenable for cellular therapy applications, would be to use programmable nucleases to promote HDR. In particular, CRISPR-Cas9 has the distinct advantage of being able to induce multiplexed cleavage^14^, simply through the inclusion of several targeting guide RNA (gRNA) sequences, which enables HDR mechanisms to potentially replace long genomic regions with an exchange cassette^15^.

We rationalized that the Major histocompatibility complex (MHC) locus would serve as a relevant proof-of-concept to demonstrate the potential for nuclease-mediated genomic exchange of immune alleles. In immunity, the highly diverse MHC locus educates and activates adaptive immunity by presenting foreign peptides from invading pathogens to guide adaptive immune responses. Yet this protective response is often detrimental during transplantation as the host MHC complexes can present, and respond vigorously to, allogeneic peptides that are derived from the donor MHC molecules^16^,^17^. The precise matching of MHC alleles between donor and recipient is therefore critically important to ensure long-term survival of donated cells. However, many clinical procedures, such as hematopoietic stem cell (HSC) transplantation for malignant (e.g., leukemia, lymphoma) and non-malignant disorders (e.g., severe combined immunodeficiency disorder), suffer from a sparse availability of correctly matched donors^18^. Several *ex vivo* genome editing approaches have been explored to mitigate MHC mismatches and generate more compatible or even “universal” donor cells. Previous studies of MHC-gene editing have been simply related to knockout; for example, ZFNs have been used to knockout the conserved MHC subunit, beta-2 microglobulin^19^, or the MHC allele directly^20^, both of which resulted in an elimination of surface expression of the MHC protein. These MHC-knockout cells are promising as “off-the-shelf” therapies but activation of downstream adaptive immune responses is compromised by the deletion of MHC alleles. To date, there have been no previous reports of MHC allelic exchange or replacement. Therefore, instead of MHC knockout, a strategy for replacement of MHC alleles would be more beneficial for improving donor-recipient matching because full immunological functionality is maintained.

As a first step towards MHC reprogramming, we used CRISPR-Cas9 to mediate rapid and scarless exchange of entire MHC-I alleles at the native locus (H2-K1) in murine-derived antigen-presenting cell lines (RAW264.7 macrophages, CT26 fibroblasts). Multiplex targeting of Cas9 with two gRNAs was used to introduce DSBs flanking the MHC-I allele, enabling HDR-mediated replacement with a MHC donor cassette (provided as a double-stranded DNA template). Thus, CRISPR-Cas9 assisted cassette exchange (CACE) was able to effectively replace an endogenous MHC-I allele (H2-Kd) of ~3.4 kb with a highly similar orthogonal allele (H2-Kb). The altered cellular phenotype was validated by the surface expression of the new MHC alleles, thereby allowing detection and facile recovery of correctly modified cells by fluorescence-associated cell sorting (FACS). The MHC-reprogrammed cells displayed a phenotype similar to a wildtype H2-Kb+ antigen-presenting cell line (JAWSII dendritic cells), while also being able to present a H2-Kb-specific antigenic peptide and activate cognate CD8+ T cells. Additionally, we investigated the efficiency of HDR with different MHC donor construct designs such as linear, plasmid, minicircle, and self-linearizing minicircle templates. We found that self-linearizing minicircle templates were the most efficient format for MHC exchange. Our approach shows the feasibility of replacing large MHC alleles at the native locus and suggests future utility for the correcting of MHC mismatches in allogenic cellular transplantations.

## Materials and Methods

All plasmids and primers used in this work are described in Supplementary Table S1.

### Cell culture

All cell lines were grown at 37 °C under a 5% CO_2_ atmosphere. Immortalized H2-Kd^+^ murine RAW264.7 macrophages (Cell Lines Service), and all subsequently modified cells, were cultured in RPMI media (61870-010; Life Technologies (LT)) containing 10% fetal bovine serum (FBS) (LT), 100 μg/ml Streptomycin and 100 U/ml Penicillin (P/S) (LT). H2-Kb^+^ JAWSII immature dendritic cells (ATCC) were maintained in MEM (32571-028; LT) supplemented with 20% FBS, P/S, and 5 ng/ml GM-CSF (PeproTech). The B3Z T cell hybridoma line was obtained as a gift from Professor Ossendorp (Leiden University Medical Center, Netherlands) and Professor Shastri (UC Berkeley, USA)^21^. These cells were cultured in IMDM media (21980-032; LT) containing 8% FBS, P/S, 50 μM β2-Mercaptoethanol (Sigma-Aldrich) and 500 μg/ml Hygromycin (LT). The CT26 cell line was obtained as a gift from Dr. Lantow (University Hospital Regensburg, Germany) and cultured in RPMI-1640 (A10491-01, Thermo Fisher Scientific) with 10% FBS and P/S supplemented.

### MHC-I locus sequencing

Unless otherwise specified all primers were ordered from Integrated DNA Technologies (IDT). Genomic DNA was extracted from RAW264.7 and JAWSII cells (1 × 10^6^ each) by the addition of 100 μl QuickExtract solution (Epicentre) according to the manufacturers instructions. PCR using the Kapa2G Fast Readymix (Kapa Biosystems) and with primers p11 and p12 was performed to amplify ~4300 bp of DNA at the H2-K locus. Primers p13 and p14 were used to amplify a backbone fragment from pUC19 plasmid [New England Biolabs (NEB)]. Gibson cloning master mix (NEB) was used to combine the respective fragments to create the storage plasmids pUC19-H2-Kb and pUC19-H2-Kd, later used for Sanger sequencing. Additionally, ~1 kb regions 5′ and 3′ to the H2-Kd locus were amplified using primers p15-p16 and p17-p18, respectively and Sanger sequenced.

### Preparation of CRISPR-Cas9 plasmids and HDR donor templates

Experiments with CRISPR-Cas9 relied on the plasmid pSpCas9(BB)-2A-GFP (pX458), obtained as a gift from Feng Zhang (Addgene plasmid #48138)^22^.The CRISPR design tool (http://crispr.mit.edu^23^) was used to identify candidate gRNA sequences near the 5′ and 3′ regions of H2-Kd gene. 5′ phosphorylated primers (guide #10 p19, p20; guide #11 p21, p22; guide #12 p23, p24; guide #13 p25, p26) were hybridized and ligated into px458 on BbsI sites to form px458-G10, px458-G11, px458-G12 and px458-G13. Donor templates were cloned into the minicircle production plasmid pMC.BESPX-MCS1 (System Biosciences)^24^. The plasmid pMC.BESPX-ET1 was created by first PCR-amplifying the H2-Kb allele from JAWSII genomic DNA with primers p27 and p28 using Kapa HiFi Hotstart 2x Readymix (Kapa Biosystems). This fragment was combined with 5′ and 3′ homology arms PCR-amplified from RAW264.7 derived DNA (5′, amplified with primers p29 and p30, and 3′, amplified with p31 and p32) and a second PCR-amplification step was performed with primers p29 and p32 to create the complete donor template. The assembled donor template was combined, by Gibson cloning, with a fragment of pMC.BESPX-MCS1 generated with primers p33 and p34. The self-linearizing plasmid pMC.BESPX-ET2 was Gibson assembled using the ET1 product from above and the pMC.BESPX-MCS1 backbone (generated with primers p35 and p36). Expected exon sequences were confirmed by Sanger sequencing of the resulting plasmids.

### Determination of CRISPR cleavage sites at MHC locus

Typically, 2 × 10^6^ RAW264.7 cells were recovered from 100 mm cell culture plates using Accutase solution (LT) and washed once with Opti-MEM media (LT). All spin steps were performed at 90 g for 5 min. Electroporation of RAW264.7 cells was performed with the Nucleofector 4D (program DS-136, Lonza). 5 μg of each of the various px458 plasmids was diluted into a volume of 100 μl SF buffer (V4XC-2024, Lonza) prior to electroporation. Following electroporation, the cells were plated in 100 mm culture dishes containing 10 ml of RPMI growth media for 24 h. After dissociation with Accutase solution, FACS (Influx, BD Biosciences) was performed to isolate Cas9^+^ cells based on 488 positive fluorescence (GFP), cells were returned to culture for 6 days. The cells were removed from the plate, washed once in PBS and genomic DNA was recovered from 1 × 10^6^ cells using 100 μl QuickExtract solution. Small fragments of DNA covering the putative cleavage sites were amplified from the genomic DNA using primers p1 and p2 for CRISPR guides #10 and #11, p3 and p4 for guides #12 and #13. For each primer set, control DNA was also amplified from wildtype RAW264.7 genomic DNA. CRISPR-Cas9 cleavage of the genome was determined using a Surveyor Mutation Detection Kit (IDT). Following hybridization of 600 ng of fragment DNA in the presence of 1 x Thermopol buffer (NEB), 1 μl of surveyor enzyme was added in a 15 μl final volume and digested for 40 min at 42 °C. All samples were run on 2% agarose gels (Sigma-Aldrich) for the detection of cleavage products. In order to test deletion of the MHC gene at the native locus, RAW264.7 cells were nucleofected as above with 2.5 μg of each of the px458-G10 and px458-G13 plasmids. Primers flanking the H2-Kd locus (p1 and p4) were used for amplification with Kapa HiFi Hotstart 2x Readymix. PCR products were run on 1% agarose gels.

### Creation of MHC-exchanged RAW264.7 cell line

Linear donor templates were amplified from a pMC.BESPX-ET1 plasmid stock using Kapa HiFi Hotstart 2x Readymix with primers p21 and p24. 25 cycles of amplification were performed with an annealing temperature of 64 °C and an extension time of 2 min. DNA was purified over Zymospin II columns, eluted in ddH_2_O, and concentrated to ~1 μg/ml using a Concentrator 5301 (Eppendorf). 5 μg of this plasmid was mixed with 5 μg of the linear donor template and nucleofected into RAW264.7 cells. After 24 h of growth, Cas9 (GFP)-positive clones were isolated by FACS and cultured for an additional 6 days in 6-well plates containing 3 ml of complete media. The media was changed once after 3 days of growth. Cells were labeled with 1 μM SIINFEKL peptide (eBioscience) in complete media for 30 mins followed by 1.25 μg/ml anti-H2-Kd-PerCP-efluor 710 (Clone SF1-1.1.1, eBioscience), and 1.25 μg/ml anti-H2-Kb-SIINFEKL-APC (Clone 25-D1.16, eBioscience, USA). FACS was used to isolate single-cell clones that were negative for H2-Kd expression and positive for high H2-Kb-SIINFEKL expression. Single-cells were grown in 96 well plates containing 100 μl complete media. To confirm transcript expression of the new allele at the H2-K locus, mRNA was isolated from each of the single-cell sorted clones (C4, F4, F5, and G5) and parental RAW264.7 and JAWSII cells using 200 μl of TRIzol^®^ reagent (LT). The mRNA was purified using a Direct-zol MiniPrep kit according to the manufacturers instructions (Zymo Research). First-strand cDNA was generated from mRNA with Maxima reverse transcriptase (LT) and used for subsequent PCRs. Allele specific primers that bridge the exon 3 – exon 4 junction for both the H2-Kb allele (p7 and p8) and the H2-Kd allele (p5 and p6)^25^ were used with Kapa HiFi Hotstart 2x Readymix to amplify small DNA fragments for detection by agarose gel and Sanger sequencing.

### Genomic characterization of MHC exchanged RAW264.7 cell lines

To determine whether integration of the H2-Kb allele occurred at the correct locus, PCR was performed on genomic DNA with primers p9 and p29, which lie outside of the 5′ homology arm and within exon 3 of H2-Kb, respectively. Evaluation of the presence of remaining H2-Kd was also analyzed by PCR using primers p5 and p6. Finally deletion at the locus was evaluated by PCR as above using p1 and p4. For sequencing of the entire H2-K locus, fragments of ~8 kb in size were PCR-amplified from the genome with primers p37 and p38 using LongAmp Taq DNA polymerase (NEB). For each PCR, the reaction was split between a minimum of 5 separate tubes and then pooled for subsequent steps. Gibson cloning was used to clone these fragments into pUC19 storage plasmids (amplified with p39 and p40), and Sanger sequenced performed using primers p41, p42, p43, p44, p45 and p46.

### Cytokine influence on H2-K allele expression

Approximately 5 × 10^5^ JAWSII and 2.5 × 10^5^ RAW264.7 cells were cultured in 24-well plates in 500 μl of complete media. After overnight growth, varied concentrations of *E.coli* 0111:B4 LPS (Sigma-Aldrich) and IL-4 cytokine (Sigma-Aldrich) were added and the culture was continued for a further 24 h. Adherent cells were recovered using Accutase solution and stained with 1.25 μg/ml anti-H-2kd PerCP-efluor 710 (Clone SF1-1.1.1, eBioscience), 5 μg/ml anti-H2-Kb FITC (Clone AF6-88.5.5.3 FITC, eBioscience), 1.25 μg/ml anti-mouse CD86 efluor 450 (Clone GL1, eBioscience) and 0.3 μg/ml anti-mouse CD80 APC (Clone 16-10A1, eBioscience). Flow cytometry (Fortessa, BD Biosciences) was used to analyze relative expression as compared to unstimulated controls.

### T cell activation assay

Approximately, 2.5 × 10^5^ JAWSII and 1.25 × 10^5^ RAW264.7 cells were cultured in 96-well plates in 200 μl of complete media supplemented with 10 μg/ml *E.coli* 0111:B4 LPS and 10 ng/ml IL-4 cytokine. After overnight culture, samples (except for controls) were incubated with 1 μM SIINFEKL peptide in complete media for 1 h at 37 °C and then irradiated with 20 Gy in a Cellrad irradiator (Faxitron, USA). 1 × 10^5^ B3Z hybridoma cells were added to each well and the plate was spun briefly at 250 g to settle the cells. The plate was incubated overnight at 37 °C with 5% CO_2_ before the cells were transferred to 96-well U-bottomed plates and spun at 2000 rpm for 2 min. The cells were washed twice with PBS and lysed with 100 μl lysis buffer containing PBS with 0.5% Nonidet P40 (Sigma-Aldrich) and 0.15 mM Chlorophenol Red-ß-D-galactopyranoside (Sigma-Aldrich)^26^. The assay was incubated for a further 6 h at room temperature with absorbance readings at 570 nm recorded every 10 min in an Infinite Pro M200 plate reader (Tecan). B3Z cells cultured alone were included as a control.

### Preparation of MHC linear and minicircle donor templates

A MHC linear donor template was amplified from pMC.BESPX-ET1 plasmid DNA using Kapa HiFi Hotstart 2x Readymix as above. To obtain minicircle DNA, single colonies from pMC.BESPX-ET1 and pMC.BESPX-ET2 were selected from fresh plates and grown for 2 h at 30 °C in LB medium (BD Biosciences) with Kanamycin. 200 ml of TB medium (Affymetrix) containing Kanamycin was inoculated with 2E-4 O.D_600_ equivalents and grown for up to 20 h at 30 °C. If the pH did not exceed 8, and the optical density was between 4 and 6, the culture was induced with 200 ml LB modified to include 40 mM NaOH and 0.02% Arabinose and grown for a further 5.5 h. Midiprep columns (Zymo) were used to purify the minicircles and the concentration was normalized after concentration measurement with NanoDrop (Thermo Fisher). 1 μg samples of each minicircle, pre- and post-induction, were digested with FastDigest EcoRI (LT) and run on a 1% agarose gel to confirm expression.

### Creation of MHC exchanged CT26 cell line

A Nucleofector 4D (Lonza, USA) was used to electroporate 2.5 μg of each of the px458 CRISPR guide plasmids px458-G10 and px458-G13, along with 5 μg of pMC-BESPX-ET2 minicircle product, in a volume of 100 μl SE buffer into CT26 cells with program CM-150. After 24 h of growth, Cas9 (GFP)-positive clones were isolated by FACS and plated in 6-well plates containing 3 ml of complete media for an additional 6 days. Bulk cells were harvested using QuickExtract solution for PCR analysis of integration with primers p9 and p10.

### Statistical analysis

For pairwise comparison between experimental groups in the B3Z assay, student T-tests were performed using the R software package. In each case a Bonferroni correction was applied to account for multiple comparisons. For comparison of donor template designs an ANOVA test was applied, taking into account batch effects, and once again accounting for multiple comparisons with a Bonferroni correction.

## Results

### Characterization of CRISPR-Cas9 target sites in the MHC locus

**Figure 1:**
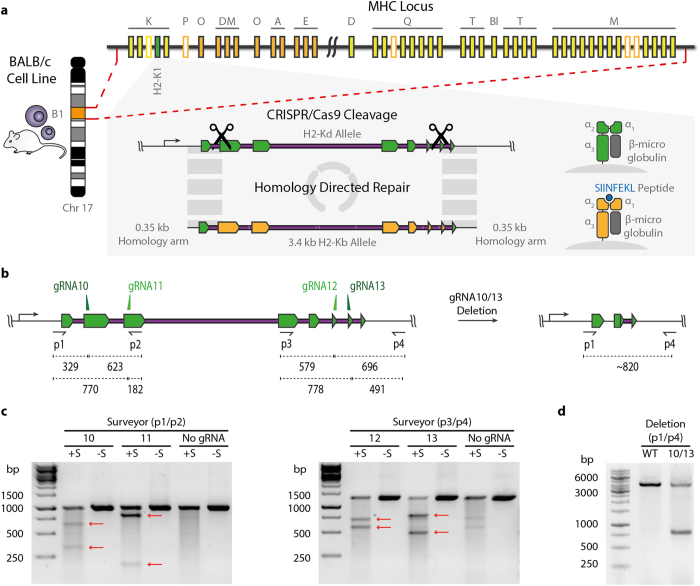
CRISPR-Cas9 targeting of the MHC locus. (**a**) Overall schematic of the experimental approach for the exchange of MHC alleles by CACE. Immortalized RAW264.7 cells carrying the H2-Kd MHC I allele are cleaved by Cas9 in the presence of a donor template containing the 3.4 kb H2-Kb allele. HDR mediates seamless exchange of the template allowing for the presentation of new peptides (e.g., SIINFEKL) in the MHC complex. (**b**) The location of gRNAs in H2-K1 locus, exons are highlighted in green and PCR primers used for amplification of the cleaved loci are shown as black arrows. The size in bp for expected cleavage products are indicated for each guide site. Deletion at the locus is detected with the flanking primer pair p1/p4. (**c**) Surveyor assay cleavage products following electroporation of cells with CRISPR-Cas9 plasmid (px458) with corresponding gRNA (10–13). Fragments were run with (+S) and without (-S) the addition of Surveyor enzyme. Red arrows indicate the detected cleavage products. (**d**) Agarose gel of genomic PCR products from RAW264.7 cells transfected simultaneously with px458-G10 and px458–G13 show deletion at the H2-K1 locus.

Murine RAW264.7 macrophages, a well-characterized antigen-presenting cell line derived from the Balb/c mouse strain, offer a suitable model system to evaluate reprogramming in the highly polymorphic MHC locus. Our overall strategy was based on using CRISPR-Cas9 to generate two DSBs in the native MHC-I H2-Kd gene while also providing a replacement donor template containing an orthogonal MHC-I allele of H2-Kb (derived from the C57BL/6 mouse strain) and homology regions flanking either side of the DSBs (Fig. 1a). HDR would then facilitate the exchange of the >3 kb MHC alleles. No selection marker (i.e., fluorescence reporter gene or antibiotic resistance gene) is required to be incorporated into the HDR donor because cells surface express MHC, thus fluorescence labeling with H2-Kd- and H2-Kb-specific antibodies can be used for selection. In order to find gRNA sites in the MHC locus of RAW264.7 macrophages, we first amplified and Sanger sequenced the H2-Kd gene and corresponding flanking regions of up to 1 kb. The exons of the H2-Kd allele share 91% nucleotide (nt.) identity with those of the H2-Kb allele from the C57BL/6 genome, with the most diversity located in the peptide binding regions encoded in exons 2 and 3 (Supplementary Fig. S1a). The CRISPR design tool (http://crispr.mit.edu^23^) was used to identify gRNA sites (compatible with *S. Pyogenes* Cas9 and its protospacer adjacent motif (PAM, 5′-NGG)) in the sequenced H2-Kd gene and the C57BL/6 genome was used as a reference for off-target events (a reference BALB/c genome was not available). Due to the inherent high degree of similarity between MHC genes, finding unique gRNA sites in this locus is especially challenging. We identified a selection of potentially H2-Kd-exclusive gRNAs sites within the exons at the 5′ and 3′ portion of the gene (Fig. 1b). To minimize the size of the required donor template, we limited the search for gRNA sites to the region between exons 2 and 7. Thus, we were able to exclude exon 1 as a target as it encodes the MHC signal peptide that is cleaved upon expression, and exon 8, as it shares 100% nt. identity between the two alleles. Each gRNA was cloned into the CRISPR-Cas9 plasmid pX458 (pSpCas9(BB)-2A-GFP), where Cas9 and green fluorescence protein (GFP) are expressed under the control of the same promoter, but as two separate proteins due to the self-cleaving T2A peptide^22^. The resulting pX458 plasmids were individually electroporated (nucleofection protocol) into wildtype RAW264.7 cells; at ~24 h, cells expressing Cas9 (via 2A-GFP) were isolated by FACS and expanded. The activity of Cas9 at each gRNA site was determined by measuring NHEJ via Surveyor nuclease assays^27^. We observed strong Cas9-induced cleavage (with the expected fragment sizes) at each of the gRNA sites tested (Fig. 1b,c). Based on their strong NHEJ activity, gRNA10 and gRNA13 were evaluated for multiplexed targeting. After simultaneous electroporation with pX458-G10 and –G13, deletion of the genomic region in between these two gRNA was verified by PCR (Fig. 1d).

### Reprogramming MHC specificity in RAW264.7 macrophages

**Figure 2:**
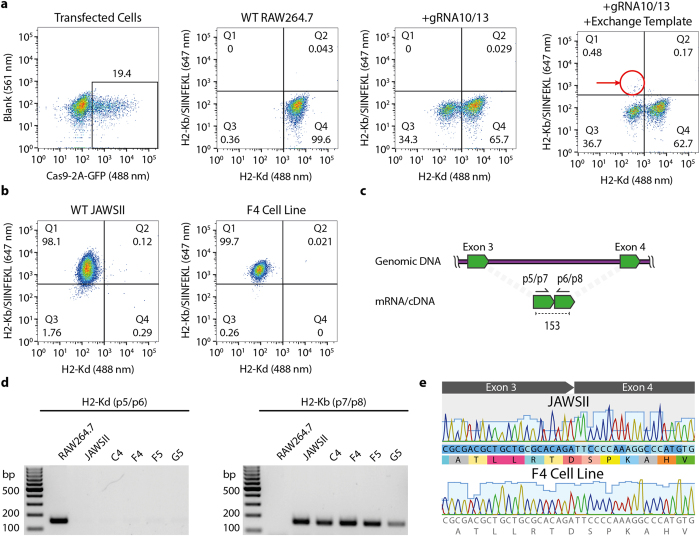
Generation and characterization of MHC-reprogrammed RAW264.7 cells. (**a**) Representative flow cytometry dot plot shows cells 24 h after electroporation with px458-G10, px458-G13 and linear donor H2-Kd template, cells were sorted for Cas9-2A-GFP (488 nm) expression (Far left). Representative flow cytometry dot plots show expression of H2-Kd and H2-Kb/SIINFEKL. Cells electroporated with px458-G10, px458-G13 and linear donor H2-Kb template show a population of H2-Kb positive cells in the Q1 gate, these cells were single-cell sorted (Far right). (**b**) Representative dot plot shows the single-cell sorted F4 clone is negative for wildtype H2-Kd expression and positive for H2-Kb/SIINFEKL expression, while showing a similar H2-Kb/SIINFEKL expression level of control JAWSII cells. (**c**) The location of primer sets used to interrogate the presence of mRNA derived from both H2-Kd (p5/p6) and H2-Kb (p7/p8) alleles. (**d**) Agarose gels show PCR products from mRNA, indicating expression from the H2-K locus for both H2-K alleles. RAW264.7 (H2-Kd+) and JAWSII (H2-Kb+) cells were used as controls. (**e**) Sequencing of the generated PCR product from clone F4 shows correct splicing of exons 3 and 4 that are separated by a ~1700 bp intron.

To reprogram the MHC H2-Kd allele of RAW264.7 cells, a ~4 kb exchange template was constructed based on the H2-Kb allele (exons 2–7), which was derived from genomic DNA of the JAWSII cell line (originating from the C57BL/6 mouse strain). The H2-Kb region was flanked by left and right homology arms (~350 bp each) corresponding to RAW264.7 BALB/c genome (Fig. 1a). In order to prevent Cas9 cleavage of the exchange template, the PAM site was modified (NGG > NGA) or silent mutations were created within the gRNA recognition sequence (Supplementary Fig. S1b,c). The donor template, generated by PCR and thus in a linear format, was electroporated with pX458-G10 and pX458-G13 plasmids into RAW264.7 macrophages. After ~24 h, cells were isolated by FACS for Cas9 expression (via 2A-GFP) and expanded in culture. In order to detect cells that had undergone MHC replacement we used a flow cytometry-based assay that relied on differential monoclonal antibody labeling of H2-Kb and H2-Kd (Supplementary Fig. S1d). To further improve the specificity of detection for H2-Kb expression, we used a monoclonal antibody that had specificity for the H2-Kb allele only when bound to a cognate peptide derived from ovalbumin (OVA_257 – 264_ or SIINFEKL)^27^. This peptide bound selectively and with high affinity to the H2-Kb MHC allele but not the H2-Kd allele (Supplementary Fig. S1d). In RAW264.7 cells that received pX458-G10 and –G13 plasmids alone (without donor template), we observed substantial knockout of the endogenous MHC gene, as 34% of cells no longer surface expressed H2-Kd (Fig. 2a). When the exchange template was included, an additional small population of cells (0.48%) appeared which were positive for H2-Kb-SIINFEKL and negative for H2-Kd. Single-cell clones were sorted from this population and expanded for further analysis.

Flow cytometry analysis on several of the single-cell colonies revealed high expression levels of the new H2-Kb protein with no detectable expression of native H2-Kd. The reprogrammed RAW264.7 cells expressed H2-Kb at similar levels to that of JAWSII dendritic cells (Fig. 2b and Supplementary Fig. S1e). To further confirm expression of the newly introduced H2-Kb allele, total mRNA was isolated and used to generate cDNA from each of the single-cell derived clones. The resulting cDNA was used in a PCR reaction with primers flanking exons 3 and 4 of the H2-Kb and H2-Kd genes, respectively (Fig. 2c). PCR products of 153 bp should only be generated if successful splicing of exons 3 and 4 occurs. All reprogrammed cell lines showed robust expression of H2-Kb transcripts, while residual H2-Kd transcripts were not detected (Fig. 2d). Sanger sequencing of these PCR products verified correct allelic expression and showed the correct splicing junction sequence for each clone, matching that of wildtype H2-Kd expression in JAWSII cells (Fig. 2e).

### Reprogrammed MHC cell lines activate T cells

**Figure 3:**
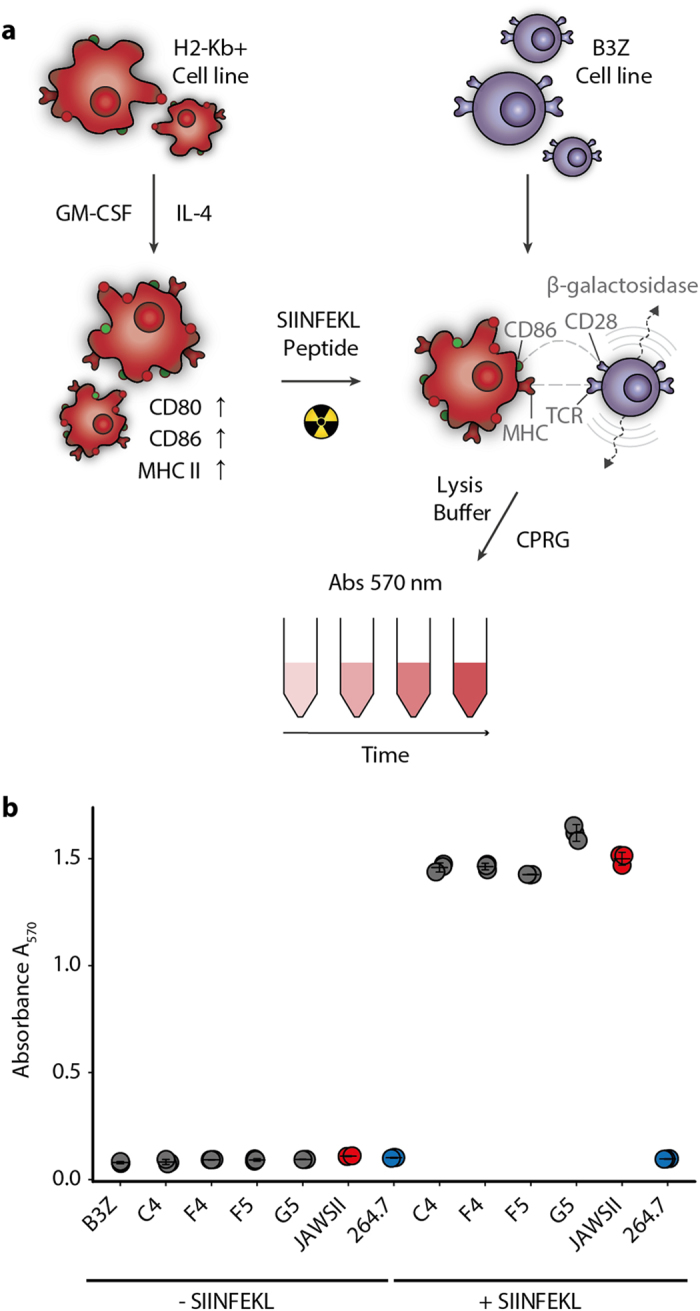
MHC-reprogrammed RAW264.7 cells activate T cells. (**a**) Experimental design of CD8+ T cell activation assay. Cell lines are activated with IL-4 and GM-CSF cytokines overnight, irradiated and combined with the B3Z CD8 T cell hybridoma cell line, which was engineered to express β-galactosidase upon TCR activation by H2-Kb/SIINFEKL peptide-MHC complexes. The cells are lysed and the amount of expressed β-galactosidase is detected by colorimetric turnover of CPRG substrate. (**b**) Representative plot of three independent experiments of B3Z activation by control and MHC-reprogrammed cell lines. The individual clones C4, F4, F5, and G5 were grown from single cells sorted from a bulk transfection. Each cell line was tested with and without SIINFEKL peptide and the absorbance at 570 nm measured after 3 hours of incubation (N = 3). Error bars indicate 95% confidence intervals for technical replicates.

We next confirmed that the newly expressing MHC H2-Kb alleles in reprogrammed RAW264.7 cells were capable of providing functional immune activity. To upregulate MHC expression and ensure a mature cell phenotype, LPS and IL-4 cytokines were added to cultures for 24 hours. Flow cytometry analysis revealed a moderate upregulation (~2-fold) of the maturation markers CD80 and CD86, while there was a nearly 2-fold upregulation in H2-Kb expression with LPS (10 μg/ml) (Supplementary Fig. S2a). The addition of IL-4 (10 ng/ml) had a less pronounced but detectable increase in the expression of these markers. The matured cells were incubated with the SIINFEKL peptide (H2-Kb-specific) and X-ray irradiated to prevent proliferation. The irradiated RAW264.7 macrophages were then co-cultured with a CD8^+^ T cell hybridoma reporter cell line (B3Z) (Fig. 3a). B3Z T cells express a TCR that is specific for the MHC-peptide complex of H2-Kb-SIINFEKL, they have also been engineered to secrete β-galactosidase upon TCR engagement^21^. All of the reprogrammed RAW264.7 clones were able to induce strong β-galactosidase expression in B3Z T cells, which was comparable to the positive control of JAWSII dendritic cells (Fig. 3b and Supplementary Fig. S2b). Activation of B3Z T cells was completely dependent on the presence of SIINFEKL peptide in the co-culture. The parental RAW264.7 macrophage cells promoted very little β-galactosidase expression from B3Z cells (comparable to B3Z cells alone) indicating that the H2-Kd allele cannot serve to activate these T cells even with SIINFEKL present.

### Genotypic characterization of MHC-reprogrammed cells

**Figure 4:**
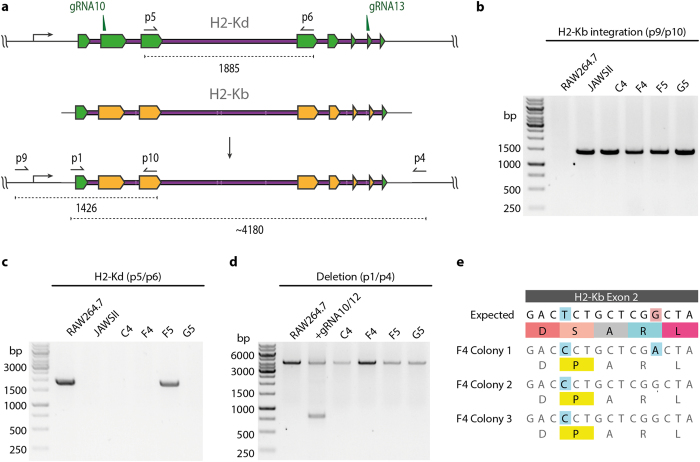
Mapping MHC allelic exchange in reprogrammed RAW264.7 cells. (**a**) Schematic of primers used for the detection of wildtype H2-Kd at the genomic locus (p5/p6) and the confirmation of H2-Kb integration at the correct locus (p9/p10). (**b**) Agarose gels show genomic PCR products that verify correct integration of H2-Kb donor cassette in all cell lines tested. (**c**) Genomic PCR analysis shows the presence of residual wildtype H2-Kd alleles only in cell line F5. (**d**) To determine whether loss of H2-Kd is likely by deletion of the allele, or by biallelic replacement; primer pair p1/p4 was used. Only the control sample shows evidence of deletion suggesting all cell lines except F5 have biallelic H2-Kd replacement. (**e**) Sequence analysis of multiple colonies from the single-cell sorted clone F4 reveals a shared template mutation.

While phenotypic analysis is able to show positive expression of H2-Kb and the lack of expression of the wildtype H2-Kd protein in reprogrammed macrophages, it is insufficient to determine whether: (i) MHC exchange occurred on both chromosomes (biallelic) or (ii) MHC exchange occurred on a single chromosome (monoallelic) in combination with NHEJ-induced knockout on the other chromosome. To address this, PCR assays on genomic DNA were designed to evaluate the various possibilities of modification in the MHC locus (Fig. 4a). In all single-cell lines tested, the newly introduced H2-Kb allele was detected by PCR (with primers p9/p10), but not in the parental RAW264.7 cell line, indicating that at least one of the alleles had been exchanged (Fig. 4b). We next looked for the presence of wildtype H2-Kd alleles, only a single colony (F5) showed a positive PCR product (p5/p6) that was comparable to the RAW264.7 positive control (Fig. 4c). This suggests cell lines C4, F4, and G5 all possessed only the new H2-Kb allele. This result correlates with the lower phenotypic H2-Kb expression observed by flow cytometry for clone F5 as compared to the other isolated clones (Supplementary Fig. S2b). However, the lack of H2-Kd allele present at the endogenous locus could either be due to exchange of the second allele or deletion at this locus. To control for this a PCR assay (p1/p4) was used to detect for H2-Kd deletion (Fig. 4d). Cells receiving only px458-G10 and px458-G13 were used as a positive control for genomic deletion. We found no bands indicating deletion in any of the cell lines tested. From each of the cell lines, we used a split-pool PCR approach to clone the inserted H2-Kb gene into a sequencing plasmid (Fig. 4e and Supplementary Fig. S3a). Each single-cell colony (C4, F4, F5, G5) had three of their resulting bacterial colonies Sanger sequenced. We observed multiple mutations that differed from the input donor template in all cell lines (Ø), and in some cases the mutations were present in all three colonies sequenced although these appeared not to have altered the H2-Kb phenotype. Because split-pool PCR was used for amplification, it is very unlikely that mutations arose during cloning into the sequencing vector. Sequencing errors are unlikely to be generated at the same location in all three colonies. Thus, mutations were most likely generated during the PCR amplification of the linear donor template despite the use of a high fidelity polymerase (KapaHiFi). Recent studies using high-throughput sequencing on PCR amplicons have shown that reproducible polymerase hotspot errors are more common than previously believed, even with high-fidelity polymerases^28^,^29^. Therefore, the generation of long donor templates by PCR may result in erroneous variants, which would present a major problem for future therapeutic applications.

### Comparison of HDR efficiency with various MHC exchange donor templates

**Figure 5:**
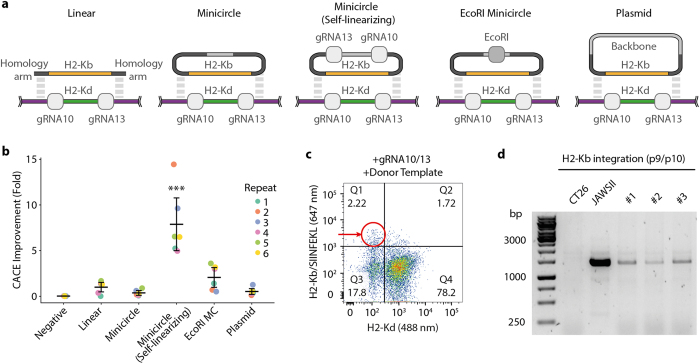
Improving MHC-reprogramming efficiency by design of alternative MHC donor templates. (**a**) Schematic of donor template designs; PCR generated linear fragments (Linear), backbone deleted minicircle DNA (Minicircle), Cas9-mediated self-linearizing minicircles containing gRNA10 and gRNA13 sites (Minicircle Self-linearizing), EcoRI pre-linearized minicircles (EcoRI Mincircle), and circular plasmid before minicircle induction (Plasmid). (**b**) Graph shows the normalized MHC-reprogramming efficiency (calculated by dividing each data point by the mean of linear template) with various donor templates. Each colored line indicates an independent experiment (N = 6). Asterisks (***p < 0.01) indicate statistical significance as compared to linear template for t-tests when corrected for multiple comparisons. Error bars indicate 95% confidence intervals. (**c**) Representative flow cytometry dotplot of a second cell line with native H2-Kd expression (CT26) following electroporation with self-linearizing minicircles and px458-G10 and px458-G13 plasmids. Cells were pre-sorted for Cas9-2A-GFP as before. (**d**) Similar to 4b, agarose gel of genomic PCR products (p9/p10) from CT26 cells shows targeted integration of H2-Kb.

To avoid PCR-based generation of donor templates, we investigated the use of alternative formats. Minicircles are a plasmid system that allows for selective removal of vector components, they rely on recombination of integrase AttB and AttP sites to remove bacterial gene elements prior to transfection in mammalian cells^24^. When compared to donor templates generated with standard plasmids, minicircles provide a smaller and less immunogenic format. We designed several minicircle donor templates and compared their exchange efficiency to our previously used PCR-generated exchange template (Fig. 5a and Supplementary Fig. S3b,c). Previous studies have suggested that linearized templates are more efficient for HDR integration when compared to circular templates^30^,^31^. Therefore, we created a “self-linearizing” minicircle exchange template, as it possessed the same gRNA sites (G10 and G13) present in the MHC locus, and thus this template would be linearized *in situ* (after transfection) by Cas9. We also included minicircles that were pre-linearized via an EcoRI restriction site. Equimolar quantities of each donor template were nucleofected into RAW264.7 cells along with the pX458-G10 and G-13 plasmids, and as before Cas9-expressing cells (via 2A-GFP) were sorted and expanded (Supplementary Fig. S3d,e). Flow cytometry analysis was used to determine the fraction of MHC-reprogrammed cells based on H2-Kb-positive/H2-Kd-negative expression. After six-independent experiments, the self-linearizing minicircle template showed the highest fold improvement in MHC exchange compared to linear template (7.9 fold on average) (Fig. 5b). No significant differences in HDR efficiency were observed for the other donor templates. PCR analysis was performed on the bulk population and revealed correct integration in the MHC locus for all samples (Supplementary Fig. S3f). To confirm the reprogramming efficiency of the self-linearizing minicircle template, we performed exchange on a second H2-Kd-expressing cell line, CT26 fibroblasts. We had previously found this cell line to be recalcitrant to MHC exchange with linear PCR templates. However, when using the self-linearizing minicircle, we observed an easily detectable level of MHC exchange by flow cytometry (Fig. 5c), which was confirmed by genomic PCR (Fig. 5d). This level of exchange was comparable to that seen with RAW264.7 macrophages, suggesting that this approach is applicable to other cell types.

## Discussion

Here we have demonstrated a proof-of-concept for reprogramming MHC-specificity in antigen-presenting cell lines using CRISPR/Cas-9 cassette exchange. In contrast to previous studies that simply knocked out MHC genes, we sought to improve MHC matching while retaining full immune functionality through the scarless replacement of MHC genes. The multiplexed capacity of Cas9 enabled the use of two gRNAs to excise the native MHC-I H2-Kd allele in RAW264.7 macrophages and replace it with a new H2-Kb allele via HDR. The surface expression of MHC proteins meant we could forgo the use of selection markers, thus MHC exchange was performed in a scarless manner, as we easily isolated cells displaying the new H2-Kb phenotype by FACS. Furthermore, the functionality of MHC-reprogrammed cells was verified through peptide antigen presentation and activation of cognate T cells. We subsequently investigated the design of the DNA donor templates and found the efficiency of exchange was significantly increased by using a self-linearizing minicircle template containing gRNA sites matching those targeted in the native H2-Kd locus. The newly designed donor template was even successful in facilitating MHC exchange in CT26 fibroblast cells, which we found previously to be resistant to exchange with linear template donors.

The downstream immune functionality of reprogrammed H2-Kb + MHC cells was validated, as they were able to engage and activate CD8^+^ T cells (B3Z cell line) in a manner indistinguishable from JAWSII cells, which are wildtype for H2-Kb. For future *in vivo* applications, biallelic scarless exchange would be preferential to monoallelic exchange combined with a knockout at the second allele. This would avoid frameshifts, which can result in the production of new out-of-frame MHC protein by-products. We tested for the presence of the original H2-Kd allele, correct integration of the new H2-Kb allele, and deletion at the locus. By this procedure we could confirm biallelic exchange was present in a majority of clones tested (3/4). As expected, cells with biallelic exchange showed higher MHC surface expression than monoalleic cells, thus suggesting phenotypic expression levels may offer a simple strategy for selection of cells with biallelic reprogramming. Upon genomic sequencing of the new H2-Kb alleles, we noted the introduction of several point mutations including some coding for amino acid changes. A split-pool PCR approach for sequencing allowed us to conclude that these mutations likely arose from the earlier PCR steps used to generate the linear donor template. To minimize the chance of mutation, we decided to test alternate donor template designs based on the minicircle technology. An important consideration when comparing plasmid-based generation versus PCR-based generation of donor templates is that the fidelity of bacterial replication is reportedly as low as 5 × 10^−10^ errors per bp, which is over 500-fold lower than the highest fidelity polymerases^32^,^33^. Furthermore, minicircles offer the advantage over plasmids in having their bacterial plasmid backbone deleted, which results in lower immunogenicity (via removal of inflammatory CpG motifs) and a smaller DNA product that can be more efficiently delivered into cells^24^. The self-cleaving minicircle donor template, which contained gRNA sites matching those used to cleave the H2-Kb allele, was the most efficient design for exchange. We propose the higher efficiency stems from kinetic reasons; the reduced intracellular processing of the minicircle compared to the linear template should allow for more donor template to be present when Cas9 expression peaks after nucelofection^34^.

Due to their critical importance in allogeneic cellular transplantation, the highly diverse alleles of the MHC locus provide a therapeutically relevant target for immunogenomic editing. Currently, intensive screening is often required in order to find suitable donors with MHC alleles that match recipients, as compatibility of multiple MHC alleles (8/8 MHC alleles matched; HLA-A, -B, -C, -DRB1) correlates strongly with transplant acceptance and long-term survival^35^,^36^. Closely related donors are generally preferred as there is a higher chance of allelic matching, although due to haplotype inheritance, even siblings will only have a 25% chance of being a perfect MHC match. Therefore approximately 50% of cellular transplantations are performed with imperfectly matched MHC donors (6/8 or 7/8 alleles matched), which often results in much higher transplantation rejection and lower survival rates^36^. Traditionally, mammalian cells have been very difficult to modify due to low rates of HDR and the reduction of proliferative capacity following delivery of the nuclease components. However, the efficiency of genome editing in these cells has dramatically improved in recent years via the development of greatly improved delivery systems for nucleic acids, most notably through the recent use of adenovirus vectors^10^,^37^. Another important consideration is the possibility of adverse events (e.g. oncogenic mutations) occurring from Cas9 off-target activity. Several strategies have been developed to reduce the off-target activity of programmable nucleases, such as the use of truncated Cas9 gRNAs, fusion of catalytically inactive Cas9 to Fok1 nucleases domains, single-stranded genomic cuts with Cas9 nickases, and engineered high-fidelity versions of Cas9^38^,^39^,^40^,^41^,^42^. In addition, the recent discovery of other programmable nucleases such as CRISPR-Cpf1 may also lead to strategies that enable high efficiency HDR with low off-target activity^43^. Moreover, the use of Cas9 as a ribonucleoprotein, rather than encoded by a plasmid, is becoming more widespread as a method to further improve the fidelity of editing^44^. All of these strategies for efficiency improvement could be readily adapted and incorporated into the approach described in this work but careful evaluation of transcriptome and proteome changes induced by replacement of the MHC locus will be required to demonstrate the specificity of the method. Likewise, while immortalized cell lines are sufficient for demonstrating proof-of-concept, our exchange methodology will need to be transitioned into primary cells for future progression towards clinical reality (e.g MHC exchanged murine bone derived macrophages co-cultured with SIINFEKL specific OT-1 primary CD8+ T cells) and subsequently towards more difficult to edit progenitor cell types such as stem cells. Careful design of *in vivo* models will also be essential to demonstrate tolerance of the engineered cells in transplantation. We hope that one day *ex vivo* MHC engineering of cells for transplantation could be very important in eliminating mismatches between hosts and donors or even applied to MHC exchange in induced pluripotent stem cell lines in order to generate cell banks for rare MHC genotypes^45^,^46^.

We thank Brian Lang and Simon Friedensohn for their aid with statistical analysis. This work was supported by the ETH Zurich Postdoctoral Fellowship (to M.P.); Swiss National Science Foundation (to S.T.R.); The National Center of Competence in Research (NCCR) Molecular Systems Engineering (to S.T.R.). The professorship of S.T.R. is supported by an endowment from the S. Leslie Misrock Foundation.

## Additional Information

**How to cite this article**: Kelton, W. *et al*. Reprogramming MHC specificity by CRISPR-Cas9-assisted cassette exchange. *Sci. Rep.* **7**, 45775; doi: 10.1038/srep45775 (2017).

**Publisher's note:** Springer Nature remains neutral with regard to jurisdictional claims in published maps and institutional affiliations.

## Supplementary Material

Supplementary Materials
