# Supplementary material for: Reprogramming MHC specificity by CRISPR-Cas9-assisted cassette exchange
Source: Sci Rep. 2017 Apr 4;7:45775. doi: 10.1038/srep45775 (PMC5379551; doi:10.1038/srep45775)
Supplement: Supplementary Materials [file srep45775-s1.pdf]

Supplementary Materials

Reprogramming MHC specificity by CRISPR-Cas9-assisted cassette exchange

Authors

William Kelton[1], Ann Cathrin Waindok[1], Theresa Pesch[1], Mark Pogson[1], Kyle Ford[1], Cristina Parola[1] & Sai T. Reddy[1], *

Affiliations

[1]Department of Biosystems Science and Engineering, ETH Zürich, Basel, Switzerland

*Corresponding author. E-mail: sai.reddy@ethz.ch

Supplementary Table 1: Plasmids and primers used in this work.

Name	Relevant characteristics or sequence (5' to 3')	Source
px458	Expression of Cas-9 nuclease and guide RNAs	[Zhang], Addgene
px458-G2	Expression of Cas-9 nuclease and guide RNA #2	This study
px458-G3	Expression of Cas-9 nuclease and guide RNA #3	This study
px458-G4	Expression of Cas-9 nuclease and guide RNA #4	This study
px458-G7	Expression of Cas-9 nuclease and guide RNA #7	This study
pMP	Expression of Cas-9 nuclease and muliple guide RNAs	This study
pMP-G2/4	Expression of Cas-9 nuclease and guide RNAs #2 and #4	This study
pMC.BESPX-MCS1	Minicircle production vector	System Biosciences
pUC19	Bacterial cloning vector	NEB, USA
pUC19-H2-Kb	Storage vector containing the H2-Kb allele	This study
pUC19-H2-Kd	Storage vector containing the H2-Kd allele	This study
pMC.BESPX-ABC	Mincircle storage vector for production of 'ABC' minicircle	This study
pMC.BESPX-ABC2	Mincircle storage vector for production of self-cleaving 'ABC' minicircle	This study
pMC.BESPX-ADE	Mincircle storage vector for production of 'ADE' minicircle	This study
pMC.BESPX-ADE2	Mincircle storage vector for production of self-cleaving 'ADE' minicircle	This study
p1 (WK406)	GAACTCAGAAGTCGCTAATCGC	IDT
p2 (WK407)	CCCTGTAATACTCTGCATCACC	IDT
p3 (WK5)	CAGGGGATGGAACCTTCCAGAAGTG	IDT

p4 (WK4)	CAGCCCTAGGTCAAGATGATAACAATCAAGG	IDT
p5 (WK202)	GCTGGTGATGCAGAGTATTACAG	IDT
p6 (WK203)	GGTGACATCAACTTGAGATCTGGG	IDT
p7 (WK200)	GCTGGTGAAGCAGAGAGACTCAG	IDT
p8 (WK201)	GGTGACTTTATCTTCAGGTCTGCT	IDT
p9 (WK89)	GGTCCGTGTGGGGCTTGCAG	IDT
p10 (WK340)	GGTAGGCCCTGAGTCTCTC	IDT
p11 (WK11)	GAACCAATCAGTGTCGCCGCG	IDT
p12 (WK12)	TAGTGACCCAGATTCTGGAAGTTTATTCATCTATC	IDT
p13 (WK24)	GATAGATGAATAAACTTCCAGAATCTGGGTCACTATTAAGCCAG CCCCGACACCC	IDT
p14 (WK25)	CGCGGCGACACTGATTGGTTCCACATTAATTGCGTTGCGCTCAC TG	IDT
p15 (WK2)	GGCGGTGACGAAATACCTCAGCG	IDT
p16 (WK8)	TCCCTCACCTCATCAGCTCA	IDT
p17 (WK7)	AATGGACAGTGATGGTGGGC	IDT
p18 (WK3)	CCTTGATTGTTATCATCTTGACCTAGGGCTG	IDT
p19 (WK369)	/5'phos/CACCGATTTCGTCACCGCCGTGTCC	IDT
p20 (WK370)	/5'phos/AAACGGACACGGCGGTGACGAAATC	IDT
p21 (WK375)	/5'phos/CACCGCGTCACAGCCGAACATCCGC	IDT
p22 (WK376)	/5'phos/AAACGCGGATGTTCGGCTGTGACGC	IDT
p23 (WK371)	/5'phos/CACCGTCCTGTCCCCACAACTAACC	IDT
p24 (WK372)	/5'phos/AAACGGTTAGTTGTGGGGACAGGAC	IDT
p25 (WK373)	/5'phos/CACCGCTGTCTCTCCCAGATGGTAA	IDT
p26 (WK374)	/5'phos/AAACTTACCATCTGGGAGAGACAGC	IDT
p27 (WK428)	CTGCTCGGCTACTACAACC	IDT
p28 (WK429)	GATCAGAGGTCTGGGAGC	IDT
p29 (WK343)	GGACGCTGGCTATAAAGTCCAC	IDT
p30 (WK430)	GGTTGTAGTAGCCGAGCAG	IDT
p31 (WK431)	GCTCCCAGACCTCTGATC	IDT
p32 (WK432)	GGCTGTGGAAGGGAAGAC	IDT
p33 (WK227)	CTCTAGAGTCGACCCATGGG	IDT
p34 (WK215)	CAGATCTGAATTCACTAGTCGCG	IDT
p35 (WK459)	GTCTTCCCTTCCACAGCCCCTTTACCATCTGGGAGAGACAGCTC TAGAGTCGACCCATGGG	IDT
p36 (WK460)	GTGGACTTTATAGCCAGCGTCCCCGGGACACGGCGGTGACGAA ATCAGATCTGAATTCACTAGTCGCG	IDT
p37 (WK116)	GGTACCCAAGTTTCAGAGGAGAGAAAC	IDT
p38 (WK132)	CGTTTGTGAAGCTGCCTAACCCCAACAG	IDT
p39 (WK129)	GTTTCTCTCCTCTGAAACTTGGGTACCCACATTAATTGCGTTGCG CTCACTG	IDT
p40 (WK130)	CCCTGAACCCCTCTTATTAGATGTTGTGATTAAGCCAGCCCCGA CACCC	IDT
p41 (WK17)	GCAGTCCTGTGCTGAGGGGAC	IDT
p42 (WK26)	GCCTGGGAGAGGCCATGGC	IDT
p43 (WK30)	CTCTCACAGCTTTTCTTCTCACAGG	IDT
p44 (WK159)	GGTCCGTGTGGGGCT	IDT
p45 (WK170)	CCTGGAACTCACTTTGTAGACCAGG	IDT
p46 (WK171)	CCTTTAATCCCAGCACTCGGGAG	IDT

13

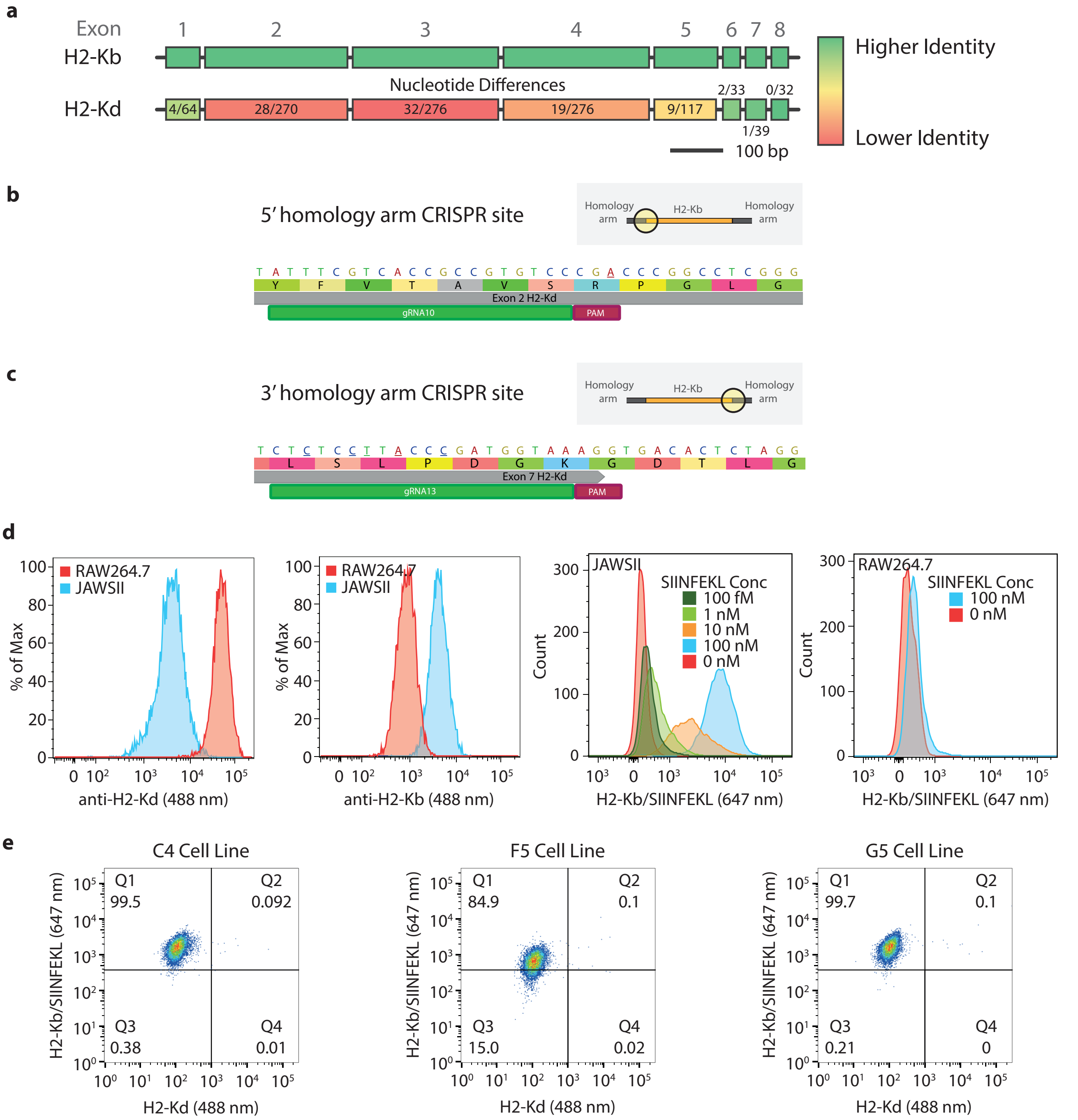

Supplementary Figure 1: Design of donor templates and selection of H2-Kb modified RAW264.7 macrophages. (**a**) Comparison of exon sequence identity at the nucleotide level for murine H2-Kb and H2-Kd MHC alleles. (**b**) The 5' homology arm junction with the H2-Kb allele; the PAM recognition site in the donor template was altered from NGG > NGA to prevent cleavage by Cas9. (**c**) The 3' homology arm junction with the H2-Kb allele; 5 non-coding mutations were introduced to alter the gRNA13 recognition site to prevent cleavage by Cas9. (**d**) In order to capture modified cells without the use of integrating antibiotic or fluorescent selection markers, we evaluated the selectivity of monoclonal antibodies against the H2-Kd and H2-Kb alleles expressed on the cell surface of RAW264.7 and JAWSII cells. Robust separation of the two cell lines was

achieved for the H2-Kd binding clone SF1-1.1.1 (PerCP-efluor 710, 1.25 μg/ml) but was not as pronounced for the H2-Kb binding clone AF6-88.5.5.3 (FITC, 5 μg/ml). We therefore titrated the amount of SIINFEKL peptide required for detection by the 25-D1.16 antibody (APC, 1.25 μg/ml) clone on JAWSII cells expressing H2-Kb and found high discrimination when 100 nM of peptide was supplied. At this peptide concentration only minimal signal was detected on H2-Kd positive RAW264.7 cells. (**e**) Additional representative FACS profiles for RAW264.7 clones isolated for H2-Kb expression following labeling for H2-Kd and H2-Kb bound with SIINFEKL.

a

RAW264.7

CD80	LPS (µg/ml)		
Il-4 (ng/ml)	0	10	30
0	1	1.9	1.9
10	1.4	1.8	1.7
30	1.3	1.7	1.7

CD86	LPS (µg/ml)		
Il-4 (ng/ml)	0	10	30
0	1.0	2.3	2.3
10	1.1	2.0	2.0
30	1.1	2.0	2.0

H2-Kd	LPS (µg/ml)		
Il-4 (ng/ml)	0	10	30
0	1.0	1.7	1.8
10	1.3	1.8	1.9
30	1.2	1.9	1.9

JAWSII

CD80	LPS (µg/ml)		
Il-4 (ng/ml)	0	5	20
0	1.0	1.2	1.3
5	0.9	1.2	1.2
20	0.9	1.3	1.2

CD86	LPS (µg/ml)		
Il-4 (ng/ml)	0	5	20
0	1.0	1.5	1.6
5	1.1	1.4	1.5
20	1.1	1.5	1.5

H2-Kb	LPS (µg/ml)		
Il-4 (ng/ml)	0	5	20
0	1.0	1.4	1.5
5	1.0	1.3	1.0
20	1.0	1.3	1.2

b

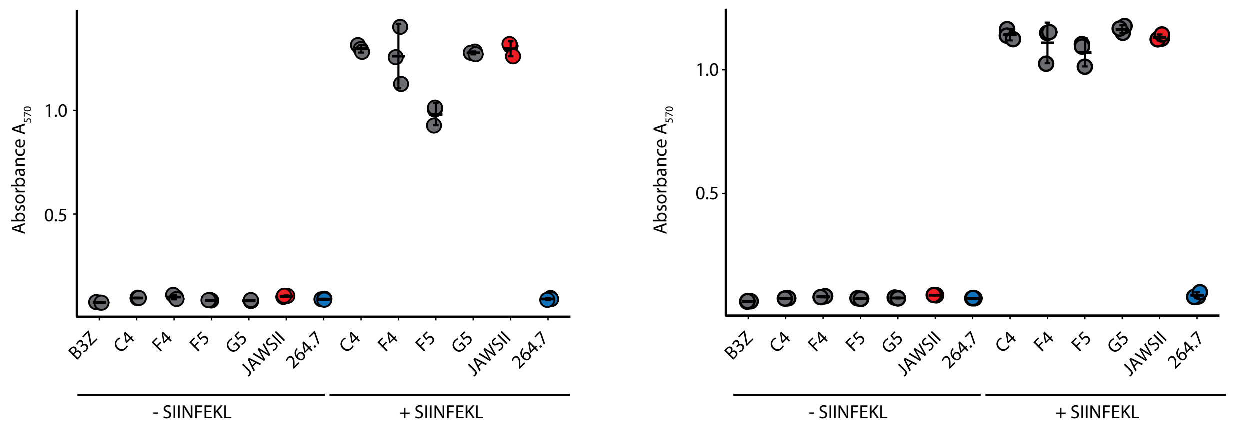

Supplementary Figure 2: B3Z T cell activation assay (**a**) Fold upregulation of antigen presenting cell markers CD80, CD86, and H2-Kb/d upon stimulation by LPS and/or IL-4 cytokine for RAW264.7 and JAWSII immortalized cell lines as determined by FACS analysis. Cells were stained with 1.25 µg/ml anti-H2-Kd (RAW264.7) or 5 µg/ml anti-H2-Kb (JAWSII) as well as 1.25 µg/ml anti-mouse CD86 and 0.3 µg/ml anti-mouse CD80. (**b**) Additional independent experiments of B3Z T cell hybridoma activation by H2-Kb+ RAW264.7 cells. Each cell line was tested with and without SIINFEKL peptide and the absorbance at 570 nm was measured after 3 hours of incubation (N = 3). Error bars indicate 95% confidence intervals.

a

Cell line	Ø	# shared	# coding
C4	9	3*	2
F4	11	3*	2
F5	8	3	2
G5	9	2	1

*mutations shared between lines

b

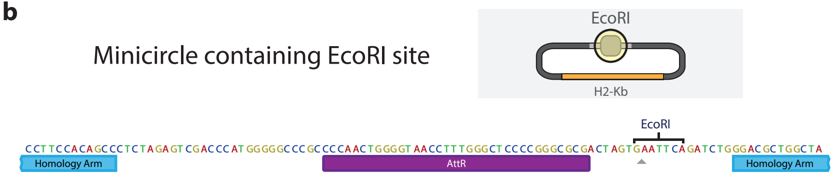

c

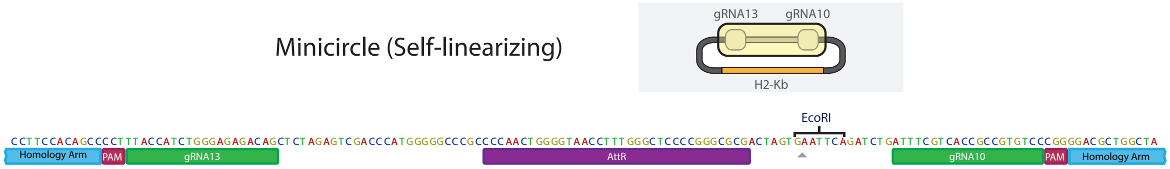

d

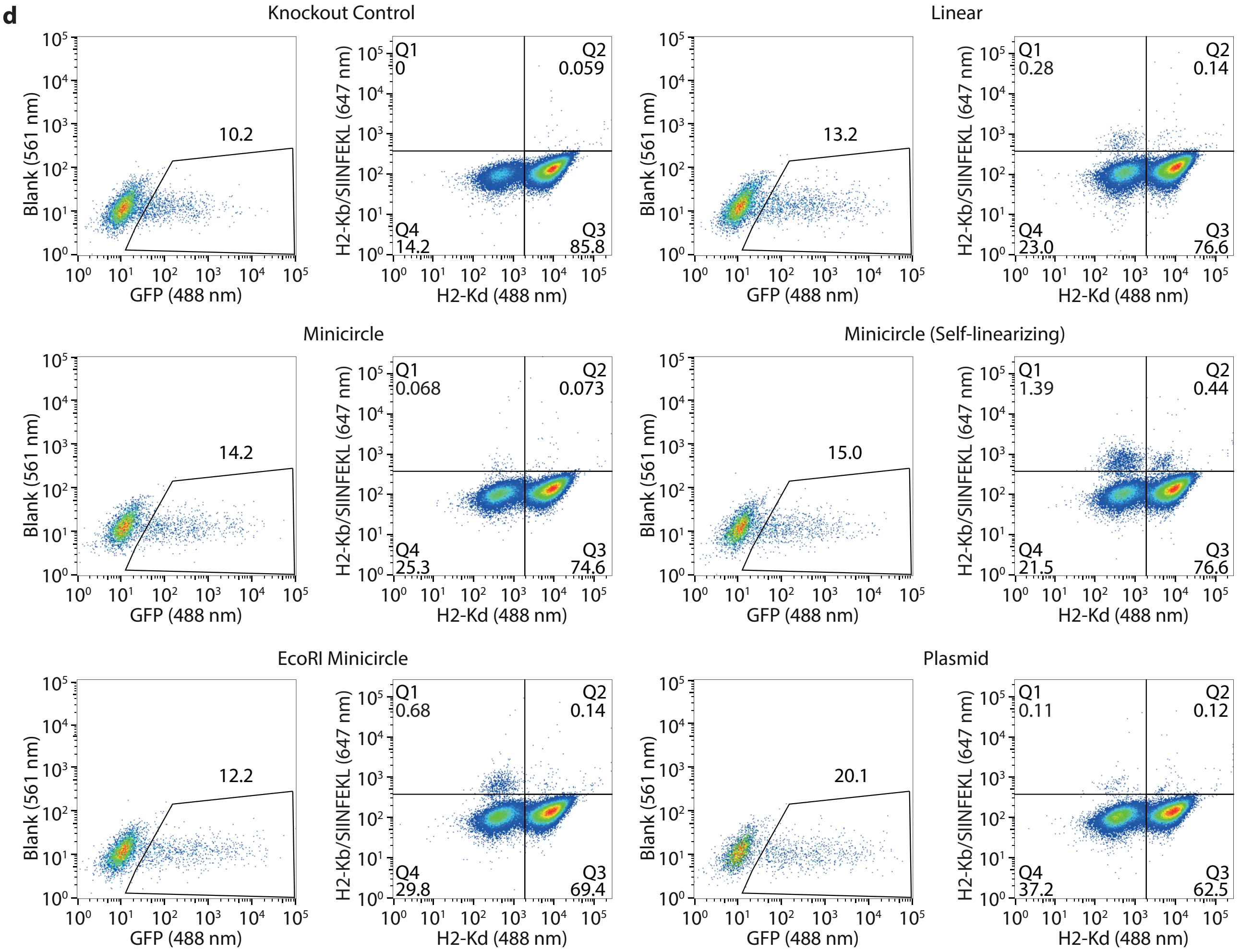

e

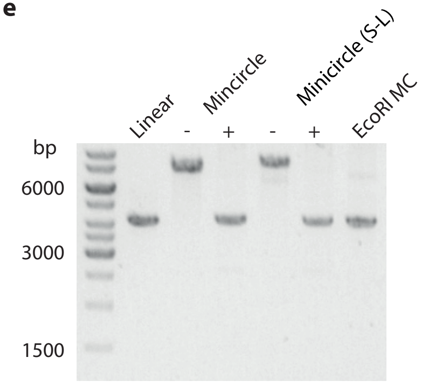

f

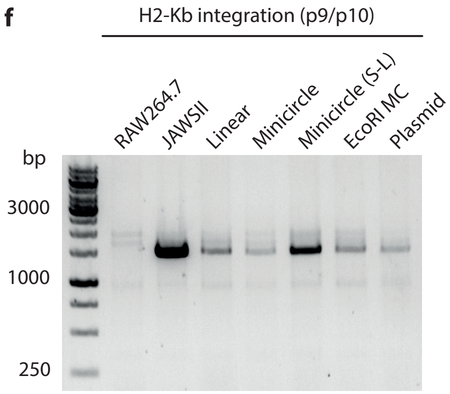

39

Supplementary Figure 3: Donor template sequencing and optimization for improved exchange efficiency. (**a**) The H2-K1 locus was amplified in all cell lines by split pool PCR and three clones were Sanger sequenced from each line. Mutations were counted in the exchanged sequence that differ from the expected donor template sequence (Ø), that are shared between the sequenced clones, and that lead to coding changes on the amino acid level. The asterisk (*) indicates identical mutations were found in the clones suggesting a common progenitor. (**b**) Expected pMC-BESPX-ET1 minicircle sequence following recombination of AttB and AttP sites. An AttR site is generated and a unique EcoRI restriction enzyme site remains to allow for linearization of the construct. (**c**) Expected pMC-BESPX-ET2 minicircle sequence following recombination showing the layout of gRNA10 and gRNA13 sites that allow for 'self-linearizing' upon expression of Cas9 in the cell. (**d**) Representative FACS data for percent allele exchange for each donor template. (**e**) Representative agarose gel of donor templates investigated for improved efficiency normalized to 1 μg/μl. Linear fragments were generated by PCR from plasmid templates pMC.BESPX-ET1 (Linear). Minicircle DNA was prepared from both pMC.BESPX-ET1 (Minicircle) and pMC.BESPX-ET2 (Minicircle Self-linearizing (S-L)). EcoRI restriction enzyme was used to create linear DNA from the pMC.BESPX-ET1 minicircle template (EcoRI MC). Plasmid DNA was uninduced pMC.BESPX-ET1 that contained the bacterial backbone (Plasmid). (**f**) Primers p9 and p10 were used to assay correct integration of the various forms of H2-Kb donor templates at the correct H2-K1 locus. Cells were sorted for GFP expression before analysis.
